# Hypothalamic–Pituitary–Gonadal Axis Involvement in Learning and Memory and Alzheimer’s Disease: More than “Just” Estrogen

**DOI:** 10.3389/fendo.2015.00045

**Published:** 2015-03-25

**Authors:** Jeffrey A. Blair, Henry McGee, Sabina Bhatta, Russell Palm, Gemma Casadesus

**Affiliations:** ^1^Department of Biological Sciences, Kent State University, Kent, OH, USA; ^2^University of Toledo School of Medicine, Toledo, OH, USA

**Keywords:** luteinizing hormone, menopause, ovariectomy, estrogen, testosterone, memory, Alzheimer’s disease

## Abstract

Accumulating studies affirm the effects of age-related endocrine dysfunction on cognitive decline and increasing risk of neurodegenerative diseases. It is well known that estrogens can be protective for cognitive function, and more recently androgens and luteinizing hormone have also been shown to modulate learning and memory. Understanding the mechanisms underlying hypothalamic–pituitary–gonadal axis-associated cognitive dysfunction is crucial for therapeutic advancement. Here, we emphasize that reproductive hormones are influential in maintaining neuronal health and enhancing signaling cascades that lead to cognitive impairment. We summarize and critically evaluate age-related changes in the endocrine system, their implications in the development of Alzheimer’s disease, and the therapeutic potential of endocrine modulation in the prevention of age-related cognitive decline.

## Introduction

As the baby boomer generation continues to age and advances in medicine have resulted in longer life expectancy, it is vital to consider the eminent risk of age-associated neurodegenerative diseases, such as Alzheimer’s disease (AD) and their effects on our society. The number of people diagnosed with AD is estimated to be over 13 million by 2050 (1). Furthermore, expense estimates for AD and dementia patient care in the United States alone are over $180 billion per year, which makes dementia one of the most costly diseases (2). Not only will more individuals continue to be diagnosed and threatened with neurodegenerative disease, but their caregivers will also suffer more physical, mental, and economic burdens.

Senile plaques containing amyloid-β, neurofibrillary tangles comprised of aggregated tau and cell death in the hippocampal formation are the hallmark pathologies of AD (3). Clinically, AD is characterized by prominent deficits in memory and attention leading to deterioration of judgment, language skills, and spatial orientation as the disease progresses (4). While AD pathogenesis has been linked to oxidative stress, inflammation, and neuronal dysfunction, concrete evidence explaining disease pathogenesis is absent, compelling us and others to search beyond the hallmark pathologies for alternative molecular cues underlying AD pathogenesis (5).

Hormones are known to impact central nervous system (CNS) function and stability (6), and gonadal hormones have been extensively studied for their effect on cognition in elderly men and women (7). We believe that age-associated endocrine system dysfunction is a major factor in the onset and progression of neurodegenerative diseases. This is supported by research showing changes in gender and age-dependent reproductive hormones increases the risk of AD, therefore implicating the hypothalamic–pituitary–gonadal (HPG) axis (8–14).

## Hypothalamic–Pituitary–Gonadal Axis

The HPG axis is controlled by a negative feedback loop. In the healthy brain, the hypothalamus releases gonadotropin-releasing hormone (GnRH) into the median eminence, and then GnRH is transported via the hypophyseal portal system to the anterior pituitary where it acts on its receptor (GnRHR). Signaling from GnRHR leads to the production and secretion of the gonadotropins, including luteinizing hormone (LH) and follicle-stimulating hormone (FSH). Once the gonadotropins are secreted into the blood stream, LH acts on its receptor in the gonads, which in turn stimulates the release of the sex steroids, androgens and estrogens. These sex steroids complete a negative feedback loop by inhibiting the release of GnRH.

Peripheral hormone levels have long been implicated in changes of behavior. Studies of the modulatory effects of hormones on cognition have led to the discovery that hormone receptors are expressed in the CNS (15, 16). Importantly, many of these hormone receptors are present in areas of the brain associated with learning and memory, such as the hippocampus. Crucial to HPG axis dysfunction and its connection to AD is that the rate of hormone synthesis varies throughout one’s life. With age, androgen production decreases gradually in men, while there is an abrupt diminution in gonadal secretion of estrogens in women. Besides, the dysregulation of the HPG axis caused by menopause may be due to a diminished ability of estrogens to inhibit the hypothalamus (17, 18). Interestingly, in addition to the negative feedback estrogens have on the HPG axis, estrogens can also produce positive feedback, which is necessary for fertility (19) and is attenuated with age (20). This body of work strongly suggests that cognitive function may rely on the levels of sex steroids and gonadotropins, implicating HPG axis dysfunction as a factor in the strong correlation between aging and dementia.

### Estrogens

Gender has been associated as an independent risk factor for AD. Females have a higher risk of developing AD as shown by age-adjusted odds ratios regardless whether familial or sporadic AD was acquired (21, 22). The higher prevalence and incidence of AD in women has been linked to the changes in reproductive hormones that occur during menopause. Therefore, women have become a main focus of many aging studies including the Women’s Health Initiative Memory Study (9, 10). Endocrinological evaluations show lower levels of estrogens in women with AD (12, 23), thus encouraging hormone replacement therapy (HRT) to improve cognition and decrease the risk of AD in post-menopausal women (24–27). This theory is based on an abundance of evidence displaying the neuroprotective effects of estrogens observed in healthy cells. Estrogens signal through a multitude of mechanisms to induce axonal sprouting, regeneration, synaptic transmission, and the prevention of cell death (28). Estrogen receptors (ER) can activate production of brain-derived neurotrophic factor (BDNF), which has been shown to protect against ischemic injury *in vitro* and retain cognitive function as measured with passive avoidance in mice *in vivo* (29). Additionally, other *in vitro* studies have shown protective effects of estrogens from excitotoxicity by increasing the apoptosis regulator Bcl-2 (30). Moreover, estrogens have been shown to protect against oxidative stress induced by amyloid-β fibrils alone or in a complex with acetylcholinesterase, making estrogens a target for AD therapeutics (31). This hypothesis is influential in that the only current AD treatment is cholinesterase inhibitors, with which meta-analyses show a modest benefit (32).

Estrogen signaling has long been known to occur through nuclear receptor activity, but can also arise through membrane-associated ERs. The classic signaling pathway relies on nuclear ERα or ERβ binding to estrogen response elements (ERE) in order to instigate transcription, therefore controlling gene expression. These long-term effects on gene expression are implicated in development as well as changes later in life. However, non-ERE-dependent signaling has been shown to rapidly produce effects of estrogens on neural processes. Membrane-associated ERs can activate PI3K/PLC, MAPK/ERK, and cAMP/PKA signaling pathways, which have been associated with neuroprotection (16). Importantly for the HPG axis, ERα mediates the negative feedback estrogens have on the hypothalamus without ERE activation to inhibit LH secretion through p21-activated kinase (33).

The neuroprotection evoked by estrogens was the basis of clinical trials looking for benefits on cognition. A randomized double-blind study found that 17β-estradiol (E2) treatment and a consequent increase in serum estrogens provided no significant improvement in cognition (34). Furthermore, the Women’s Health Initiative funded a study of 16,000 women that indicated HRT, with E2 and progestin, in fact increased risk of dementia. However, after controlling for treatment onset, it was found that initiation of HRT 10 or more years after menopause increases the risk of AD, while initiation of HRT during menopause lowers risk of AD (9, 10). Supplemental research showed the treatment group in the Women’s Health Initiative Memory Study had a higher incidence of breast cancer (35). This off target effect was pronounced with a combined E2 and progestin treatment, but is a major concern in HRT despite cognitive benefits observed. Overall, the clinical trials suggest that a critical period between menopause and HRT onset exists (36).

The results of E2 treatment have led to the proposal of the critical period hypothesis, which states that HRT is beneficial immediately after menopause, but benefits are lost the longer post-menopause is endured (36). The critical period hypothesis has also been demonstrated in ovariectomized rodents. The immediate post-operational treatment with E2 produces the well-characterized cognitive improvements, but a latency period of several months before E2 treatment ablates all benefits (37, 38). Along with the critical period hypothesis is the idea of the healthy cell bias. Estrogens may only be beneficial when administered to healthy neurons, and in order to potentially protect from neurological damage estrogens would need to be preemptively administered (39, 40). Conversely, once an unhealthy neurological state prevails estrogens signaling produces deleterious effects. Current hypotheses on the negative effects of estrogens in unhealthy neurological conditions include aberrant calcium signaling and mitochondrial dysfunction (41).

The reduced efficacy of estrogens downregulation of gonadotropins is evident in rodents after ovariectomy (42) and in post-menopausal women (43). Therefore, an alternate hypothesis that explains the lack of efficacy of HRT in older post-menopausal women is the inability of HRT to provide efficient negative feedback on gonadotropins. In this regard, LH is now known to influence cognition as well (44–48). Therefore, methodologically separating the roles of estrogens and LH on cognition is imperative to fully understand the impact of these hormones on cognitive decline and AD (37).

### Luteinizing hormone

In conjunction with estrogens function in cognition, the loss of sex steroids leads to large increases in peripheral levels of LH. In aging women, a threefold increase in LH and a fourfold increase in FSH levels occur (49) while in aging men both hormones increase two to threefold (50). Until recently, the impact of changing levels of gonadotropins during reproductive aging was virtually ignored given the primarily peripheral role of gonadotropins on reproduction. However, mounting studies in humans, rodents, and *in vitro* demonstrate that LH and activation of its receptor may have an important role on cognitive function and neuronal plasticity.

In humans, increases in peripheral LH levels are correlated with decreased cognition in both healthy women (51) and men (52), and levels of LH and FSH have been shown to be significantly increased in AD patients compared to control (8, 13) in some but not all studies. For example, serum LH and FSH levels have been shown to remain consistent in female (12) and male (53) subjects regardless of dementia status, but by increasing the sample size, Hogervorst et al. (11) observed a trend toward high serum LH correlating with AD status in males. In a recent study, LH levels were correlated with amyloid-β levels, further implicating LH in development and progression of AD (14).

Human chorionic gonadotropin (hCG) and LH share a receptor (LHCGR) crucial for reproductive functions, such as testosterone production by Leydig cells and follicular maturation. A large body of literature describes the physiological and molecular role of LHCGR in reproduction (54). The isolation and purification of LHCGR revealed that it is a member of the G-protein-coupled receptor (GPCR) family (55, 56), and part of rhodopsin-like class A GPCRs expressed as several splice variants, all of which have leucine-rich repeats in the extracellular domain (54). Upon phosphorylation of LHCGR, Gα_s_ protein activates adenylyl cyclase and ultimately stimulates the cAMP/PKA and ERK pathways. Although LHCGR primarily signals through the Gs/cAMP/PKA pathway, it can independently mediate the activation of phospholipase C [PLC; (15, 57)].

Accumulating research shows LHCGR is atypically expressed in the CNS (58–60). LHCGR transcripts of 2.5 and 4.3 kb have been shown in fetal rat brain neurons and glial cells (61, 62). An 80 kDa band as seen by western immunoblot is evidence of protein expression in cultured rat neurons. Furthermore, time course studies show that day *in vitro* (DIV) 3 neurons have the highest levels of expression of LHCGR (61). A similar 80 kDa band is seen in immunoblots from glial cells which increase with an increase glial proliferation (62). Similarly, *in situ* hybridization has shown an abundance of LHCGR in the rat brain including the hippocampal formation, hypothalamus, cerebellum, choroid plexus, and ependymal cells of the ventricles and cortex (63). LHCGR expression has also been shown in the CNS of *Xenopus laevis*, where it plays a functional role in the reproductive behavior of courtship songs, therefore implicating a non-gonadal function of LHCGR in the CNS (64). Although many laboratories have researched LHCGR mechanisms in the gonadal organs, extra-gonadal receptor processes in rodents are just now beginning to be elucidated.

For example, early work shows that direct activation of the LHCGR with hCG drives cellular and functional changes. To this end, rat neurons cultured in the presence of hCG appeared to have increased numbers of neurite-bearing cells (61). The same treatment paradigm for glial cells showed an increase in prostaglandin D2 and a decrease in prostaglandin E2 (62). Importantly, prostaglandin D2 is known to curb the proliferation of glial cells; therefore, LHCGR activation in the CNS may be an important modulator of glial cell populations (62). Cell viability was increased with hCG treatment as observed through increased total protein content and decreased DNA fragmentation (61).

At a functional level, open-field behavior on rats injected with hCG either intraperitoneally (IP) or intracerebroventricularly showed a decrease in locomotor activity, but no increase in anxiety (65). Taste neophobia was overcome by intraperitoneal injections and intracerebroventricular injections of hCG, and hCG treatment did not evoke differences on T-maze behavior for the goal box, but a decrease was observed in the stereotypic behavior (65). This study shows that at a high dose hCG affects overall activity, which will supersede any findings related to learning and memory or fear. It has been proposed that at these high doses, hCG may be responsible for changes in sleep that occur during pregnancy since hCG is present in cerebrospinal fluid during the first trimester and correlates with serum levels during the first and third trimester (65–67). IP injection of hCG in rats showed increases in both high and low amplitude sleep and a decrease in active awake phase via electroencephalography [EEG; (68)]. Similarly, peripheral hCG administration has been shown to decrease walking and increase resting in rats (68). It is important to note that during sleep LH pulses decrease in frequency but increase in amplitude (68).

Furthermore, studies using therapies aimed at downregulating peripheral LH show significant improvements in cognition and AD pathogenesis (44, 45). For example, in AD mice, downregulation of serum LH improves function in Y-maze and Morris water maze cognitive tasks and reduces amyloid-β immunoreactivity in the Tg2576 AD mouse (44). Animal studies utilizing GnRHR antagonists, antide and Cetrorelix, which also lowers serum levels of LH, also show cognitive improvements in spatial memory tasks in rats (46) and in non-transgenic models of AD (47, 69).

At a signaling level, studies show that downregulation of LH leads to activation of memory-associated cascades. CaMKII auto-phosphorylation, which is associated with hippocampal long-term potentiation (LTP), is downregulated after ovariectomy, but rescued with leuprolide acetate treatment (70). Furthermore, downstream targets of CaMKII, such as the phosphorylation of GluR1 subunit of AMPA are also activated by leuprolide acetate treatment (70). These changes in LTP-related cascades are one potential mechanism underlying the behavioral improvements observed with leuprolide acetate treatments *in vivo* (44, 48, 70). Interestingly, leuprolide acetate can also affect the synthesis of E2 from testosterone by modulating the transcription of p450 aromatase (70) suggesting functional benefits may be secondary to up-regulation of endogenous estrogens and downstream modulation of CREB and GSK3β (48, 71, 72). Taken together, how LHCGR activation influences these cascades and leads to functional changes is a critical area of future study.

Interestingly, LH immunoreactivity has been demonstrated by radioimmunoassay as well as immunocytochemistry in the hypothalamus, amygdala, septal area, preoptic area, thalamus caudate nucleus, and hippocampus (73). Extracts from rat brain have shown that brain-derived LH has a similar chromatographic profile of pituitary LH and is active in the testis LH radio-ligand receptor assay as well as the interstitial cell testosterone secretion bioassay (74). Additionally, an adsorption significantly reduced rat brain LH immunoreactivity (74). Therefore, LH is both present and biologically active in the CNS. In support of these earlier studies, we have shown LH immunoreactivity in cognition-related brain regions in the 3xTg AD mouse model. Importantly, levels of brain-derived LH are decreased by ovariectomy, a state of high peripheral LH, and positively correlated with improvements in the Morris water maze (48). This suggests an inverse correlation between serum (peripheral) LH levels and CNS LH levels. Importantly, this inverse relationship between peripheral and brain-derived LH may explain the beneficial effects of hCG seen by Al-Hader et al. (61, 62) *in vitro* as well as the functional benefits and activation of LHCGR-dependent cascades by leuprolide acetate (48, 70).

### Androgens

Hypogonadism, a common feature of aging, may significantly contribute to both physical and cognitive decline in men, including the development of AD (75, 76). It has been shown that men with AD have lower levels of testosterone compared to non-AD patients (53). However, results have been mixed concerning testosterone replacement in healthy men. Testosterone replacement in non-demented patients with healthy gonadal function increased spatial cognition, verbal memory, and working memory (77–79). In contrast, testosterone replacement studies focusing on upper extremity strength or self-perceived health did not observe changes in cognition after 1 year of treatment (80, 81). Nevertheless, patients with mild cognitive impairment or AD have benefited from testosterone replacement. Spatial memory, constructional abilities, and verbal memory have all been shown to improve in cognitively impaired patients with short- (82) and long-term (83, 84) testosterone treatment. Meanwhile, one study suggests no difference between placebo and testosterone treatment (85), but the behavioral measures used may not have been sensitive to testosterone treatment (86), and, importantly, the testosterone injections may have been too interspersed to improve cognition. Trends in the literature suggest positive correlations between testosterone treatment and cognitive improvement warranting further investigation as a viable option for treating AD.

Essential to direct effects of testosterone on cognition, the androgen receptor (AR) is expressed in neurons of the hippocampus and amygdala (87, 88). Primary neuron cultures treated with testosterone show decreases in secretion of amyloid protein (89). *In vivo* studies show increased testosterone corresponds to decreases in β-secretase, an enzyme responsible for the cleavage of the amyloid-β precursor protein, and improves cognitive function in male double-transgenic mice (90). Effects of testosterone on CA1 spine synapses are independent from the AR since similar effects are seen in normal rats and AR mutants (91). In rats, rapid acting cytoplasmic androgen signaling in axons of the dorsal CA1 hippocampal neurons innervating the amygdala may be the most likely route for learning, especially in relation to environmental cues (88).

The AR binds testosterone or dihydrotestosterone (DHT) and translocates from the cytoplasm to the nucleus. Ultimately, AR signaling modifies gene expression, but like ERs, the ARs also have effects on intracellular signaling such as DHT’s induction of CREB activation (92, 93). Extranuclear ARs are localized exclusively in asymmetric synapses (94) while ERs are found in both asymmetric and symmetric synapses (95). Redundancy may exist given the location of the ERs and ARs, but a unique function may exist for ER in symmetric synapses, which are typically inhibitory. Interestingly, for the action of ARs and ERs, testosterone can be converted into E2, which requires the experimental delineation of androgen and aromatized androgen metabolite action (96).

The conversion of testosterone into E2 by p450 aromatase (97) affords testosterone the ability to activate both ARs and ERs within the brain. Testosterone conversion can be controlled by aromatase inhibitors, therefore allowing experimental separation of testosterone pathways and the pathways of its aromatized metabolites (96). In a study of 60 healthy men aged 50–90, men treated with testosterone alone, and which consequently increases E2 levels from aromatase activity, displayed improvements in verbal memory. In contrast, when an aromatase inhibitor was administered along with testosterone, the improvement was no longer observed, suggesting that aromatization of testosterone is necessary for improvements in verbal memory (77). The individual actions of the sex steroids have separate roles in cognition; however, it is necessary to take neurological health into consideration during treatment. Testosterone metabolites improve cognition in healthy individuals, but as is evident from studies of estrogens, the neurological health of the patient may be a confounding factor.

Overall, a decrease in testosterone levels is associated with a higher risk of AD (11, 13, 14), and improvements in cognition have been observed with testosterone treatment (82–84). Testosterone signaling can occur directly through AR action or through the aromatization products acting on ERs but the bioactivity of the sex hormones may be a confounding factor (96). Increased levels of sex hormone-binding globulin (SHBG), which binds to sex steroids and reduces their bioactivity, have been linked to an increased risk of dementia in both men and women (98).

### Sex hormone-binding globulin

Confounding the age-dependent attenuation of sex steroid signaling in the HPG axis is the ability of SHBG to reduce the bioactivity of sex steroids through binding and inhibiting action on their respective receptors. AD patients have increased levels of SHBG and, therefore, lower serum levels of bioactive sex steroids. The ensuing inverse correlation between cognition and SHBG is observed in both male and female AD patients (11, 98–101). SHBG levels and its inverse correlation with cognitive decline are important to consider for determining the efficacy of E2 and testosterone treatment. Importantly, SHBG levels may confound previous studies by deactivating the sex steroid treatments. Although further research is necessary, therapeutic control of SHBG would allow endogenous estrogens and androgens to remain bioactive and potentially reduce the risk of cognitive decline.

## Conclusion

A myriad of research on the role of hormones in cognition has been undertaken and has led to many outlets for therapeutic potential to combat the cognitive loss associated with aging and AD. Epidemiological studies of the aging population show not only a gender difference in risk for AD, but also that HRT can have cognitive benefits. Prospective studies in humans and animals show that E2 treatment provides benefits to cognition when treatment onset is within the critical period, temporally close to menopause or hormone dysfunction. On a mechanistic level, it is well known that the levels of estrogens positively correlate with dendritic spine density in rodents as well as axonal sprouting, lending evidence for E2 treatment benefits being mediated through increasing plasticity. However, the diminished and deleterious effects of E2 treatment implemented outside of the critical period and the implication of this hormone in the development of breast cancer begets the search for hormone-based therapeutic strategies that go beyond E2 treatment. In this regard, one potential area of research is testosterone replacement. Work presented in this review demonstrates the beneficial effects of this hormone on learning and memory. However, effects of testosterone on cognitive function are primarily related to the aromatization of testosterone into E2. As such, while testosterone may provide advantages through its ability to signal through AR and E2 receptors after its aromatization, this also poses issues at the level of dosing and off target effects. Taken together, while the actions of sex steroids have adverse effects, the improvements observed highlight the feasibility of HRT as a pharmaceutical treatment for cognitive decline throughout aging. However, aspects such as timing of replacement and clinical follow-up to mitigate important side effects must be carefully watched. Also important is the fact that secondary players, such as SHBG and p450 aromatase, that regulate bioavailability or production of steroids are key in determining the magnitude of effect and specificity of steroid hormone treatment. Whether these secondary players have a direct impact on cognition and neuroplasticity is unclear, and their determination is particularly important when interpreting the results of clinical trials using sex steroid replacement.

Lastly, we provide evidence that LH, a relatively unknown player in cognitive function and neuronal plasticity, is gaining support as a therapeutic target for age-related cognitive decline. Support for a role of LH in cognitive processes is highlighted not only by clinical data demonstrating high levels of peripheral LH are associated with cognitive deficits and AD, but also in pre-clinical studies. To this end, several investigators have now shown that lowering peripheral LH rescues cognitive decline in various aging and AD rodent models, and these treatments are associated with signaling important to synaptic plasticity. Importantly, a recent clinical trial in AD female patients shows benefits of downregulating peripheral LH (102).

Here, we propose the hypothesis that brain levels of LH become downregulated by increases in peripheral LH observed during ovarian function loss and cause cognitive dysfunction; this aspect, we hypothesize to be mediated through loss of LHR signaling in the brain. While the mechanisms in charge of downregulating brain LH production are unknown, support for this hypothesis is emerging based on data showing LHR expression and functionality in the brain as well as receptor actions associated with cognition-related signaling and neuroplasticity. Importantly, in our recent study, we observed a positive correlation between LH levels in the brain and learning and memory in the Morris water maze task. Studies directed at elucidating the specific role of the LHR in the brain are likely to shed light on the role of LH in cognition and neuroplasticity.

While in the last 30 years primary focus has been placed on studying the role of gonadal steroids on neuronal plasticity and function, we show here that aspects thought to be secondary or irrelevant, such as LH signaling, are important to fully evaluate the effects or lack thereof of gonadal steroids, and may potentially have direct roles and drive processes previously assigned to loss of steroid function. As such, a comprehensive study of HPG axis hormones is necessary in clinical and pre-clinical work and likely to be more effective in providing novel therapeutic targets and strategies for cognitive dysfunction and AD.

## Conflict of Interest Statement

The authors declare that the research was conducted in the absence of any commercial or financial relationships that could be construed as a potential conflict of interest.
